# Phenomenal Bombardment of Antibiotic in Poultry: Contemplating the Environmental Repercussions

**DOI:** 10.3390/ijerph17145053

**Published:** 2020-07-14

**Authors:** Muthu Manikandan, Sechul Chun, Zakayo Kazibwe, Judy Gopal, Udai Bhan Singh, Jae-Wook Oh

**Affiliations:** 1 Department of Environmental Health Sciences, Konkuk University, Seoul 143-701, Korea; bhagatmani@gmail.com (M.M.); scchun@konkuk.ac.kr (S.C.); kajazacorp@gmail.com (Z.K.); jejudy777@gmail.com (J.G.); 2 Plant-Microbe Interaction & Rhizosphere Biology LabICAR-National Bureau of Agriculturally Important Microorganisms, Kushmaur, Mau Nath Bhanjan 275103, Uttar Pradesh, India; udaiars.nbaim@gmail.com; 3 Department of Stem Cell and Regenerative Biotechnology, Konkuk University, Seoul 143-701, Korea

**Keywords:** poultry, composting, microorganisms, antibiotics, degradation, environment, toxicity

## Abstract

Antibiotics have constantly been added at an unprecedented rate in order to enhance poultry meat production. Such antibiotics impose a negative impact on human health directly through meat and egg consumption. On the other hand, they also affect humans indirectly by affecting the normal key microbial processes in the agricultural environments, when used as poultry compost. For many years, farmers have been turning poultry litter into compost for agricultural use. Very few studies have addressed the fate of the unmetabolized antibiotic residues in poultry litter that could potentially affect microbial communities when used as poultry compost. We have also questioned the fate of residual antibiotic in poultry waste which may create possible negative environmental pressure on microbial communities that are involved in microbial mediated poultry litter composting processes. The incorporation of antibiotic degrading environmental isolates in poultry litter at the initial stage of composting in order to accelerate the process is addressed in this review as a future perspective.

## 1. Introduction

The poultry industry is a valuable resource for meeting the protein needs of mankind through meat and egg supply. According to the Food and Agricultural Organization (FAO) report 2018 [1], poultry industry is one of the largest contributors amidst the global meat market with an estimated quantity of about 122.5 million tons. The increasing demand as evidenced in the past, signifies the vital role of poultry products. Therefore, the United States Food and Drug Administration approved the use of antibiotics as animal feed additives without veterinary prescription from 1951 in order to meet the rising demand [2]. European Union had (~50 years before) given the green signal for the unrestricted usage of any antibiotic as growth promoters [3]. Undoubtedly, this has accelerated meat and egg production to meet the rising human needs. On the other hand, this has also led to an alarming increase in the usage of a large number of antibiotics which in turn have affected human beings either directly through consumable poultry products or indirectly through destroying the beneficial microbial population that aid in agriculture. As it is well known, poultry litter is turned into poultry compost and used for enriching the agricultural environments.

It has been estimated that 1000 mature layer chickens approximately excrete 120 kg excreta/day and meat chickens: 80 kg/day, turkeys: 200–300 kg/day and ducks, 150 kg/day [4,5,6]. Although the poultry litter, as such, contains enormous nutrient content, the manure cannot be used directly because it does not support plant growth significantly when used as such without prior pretreatment. This is owing to the presence of high (60–80%) organic nitrogen content (as protein and urea) [7]. This nitrogen cannot be assimilated by plants. Furthermore, ammonia (~40–90%) is constantly being formed from the poultry litter for a period of one year and such an accumulation causes acidification of soil substrates and vital cation (K^+^ and Mg^2+^) displacement that cause chlorosis of leaves and growth suppression [8,9].

Composting of organic wastes, in general, is considered as an oxidative process mediated by bioentities via biomineralization and partial humification of the organic matter yielding a stabilized final product with certain humic properties and without phytotoxicity and pathogens [10]. Therefore, the poultry litter should be composted for a long time to release its maximum plant growth promoting potential. This generally relies on factors like water content, bedding materials (i.e., straw, wood shavings or rice hulls as is typical in meat bird housing) and the microbial population [11]. Sweeten (1988) had achieved poultry litter composting process in 4–6 weeks to obtain a minimum-quality-stabilized material [12]. Further, improvements had been made in poultry litter composting process by the addition of various amendments that includes two natural zeolites, clay, coconut coir. CaCl_2_, CaSO_4_, MgCl_2_, MgSO_4_ and Al_2_(SO_4_)^3-^ have been added in order to reduce the composting period between days 49 to 56 for obtaining the stabilized compost containing mineralized ammonia [13].

Although the influence of many factors in poultry composting process have been studied [14,15,16,17,18,19], the role of one of the vital factors, i.e., antibiotics (which is supposed to be a determinable factor for the bio-entities that perform the biooxidative process during compost maturity), had completely been ignored. The presence of antibiotics is no longer in trace quantities but has become alarmingly high, since an enormous amount of antibiotics with broad spectrum of activity have been used in poultry. Therefore, this current review puts on journal record the significance of poultry manure and the role of antibiotics used in poultry and their impact on the microbial system that performs various biooxidation processes. While the effect of antibiotics and their pharmacokinetics in poultry meat and their system has been studied, almost no validated reports exist with respect to antibiotic residues in poultry litter and poultry compost and their subsequent movement in agricultural soils and crops and animals and humans. This review presents the future perspective of resolving this crisis through an accelerated composting process involving antibiotic degrading microbes.

## 2. Nutrient Content of Poultry Feed and Excreta

Poultry manure quality starts with the quality of the poultry feed. Therefore, in order to evaluate the nutrient content of the poultry waste, it is important to know the poultry feed composition because a considerable proportion of the feed passes through the body unabsorbed and thus reaches the poultry litter. The largest composition of poultry feed contains carbon sources from rice, wheat, sorghum and corn [20]. In addition to that, it also contains protein (nitrogen) sources from soy meal, canola, lentils and sunflower sources. Fishmeal, meat and bone meal are also important protein sources in poultry feeds [21]. Poultry feed also contains mineral supplements which include calcium from limestone, shell grit, dicalcium phosphates, defluorinated rock phosphate, bone meal, mineral premixes like sodium salt, sodium bicarbonate. Furthermore, vitamins, amino acids like methionine, lysine, threonine, enzymes and antibiotics, pesticides, acaricides and other food supplements also constitute some percentage of the poultry feeds [22].

Most of the poultry feed components pass through the animals’ gut partially or completely undigested hence the poultry litter consists of undigested nitrogen (N), phosphorus (P) and considerable amounts of other substances such as hormones, antibiotics and heavy metals [23,24]. Due to the inefficient conversion of dietary nitrogen in poultry birds, 55–80% of the total nitrogen is excreted [25,26,27]. Further, the broiler chicken also excretes approximately up to 80% of the phosphorous and 80% of the potassium and 50% to 80% of the nitrogen [28]. P is partly present in poultry diets as phytic phosphorus, i.e., a form that is poorly digestible by birds due to a lack of adequate endogenous phytase activity [23].

## 3. Significance of Poultry Manure

Uncomposted poultry litter contains a huge amount of organic carbon, nitrogen and phosphorus which rarely support the growth of crop plants. Therefore, the litter becomes usable only after composting, yielding what we mention in this review as ‘poultry compost’. Subsequent to composting for a considerable period of time, the concentration of nitrogen (3–5%), phosphorous (1.5–3.5%) and potassium (1.5–3.0%) changes leading to accelerated compost properties [29,30,31]. Further, it is also been reported to contain all the essential micronutrients for plants such as calcium (Ca), magnesium (Mg), sulfur (S), manganese (Mn), copper (Cu), zinc (Zn), chlorine (Cl), boron (B), iron (Fe) and molybdenum (Mo) at considerable levels [31,32,33]. Thus, it is clear that the composted poultry manure contains enough quantities of nutrients that can support not only crop yield but also enhance the physicochemical properties of the soil. As a matter of fact, the application of poultry manure improves the irrigation efficiency and dryness resistance of sandy soils by enhancing the moisture retention capacity and lateral water movement of the soil. This causes longstanding plant nutrient retention in soils thereby rendering extended uptake of nutrients in plants [34,35,36,37]. In addition to that, poultry manure also promotes the microbial community in the soil. These microbes support plant growth and offer protection against various plant pathogens [38,39,40].

## 4. Microbial Content of Poultry Litter

Lu et al. [41] evaluated the microbial composition of broiler litter using 16S rRNA and Functional Gene Markers. Their report revealed that *Enterococcus sp*. and Coliforms were detected by culture-based bacterial detection, while culture independent methods revealed other group of bacteria such as *Globicatella sulfidofaciens*, *Corynebacterium ammoniagenes*, *Corynebacterium urealyticum*, *Clostridium aminovalericum*, *Arthrobacter* sp. and *Denitrobacter permanens*. Other bacterial strains with pathogenic character such as Clostridia, Staphylococci and *Bordetella* sp. were also reported when poultry litter DNA samples were used as templates for microbial diversity assessment [41]. Enticknap et al. 2006 [42] had identified a variety of bacterial species namely *Nocardiopsis* sp., *Anthrobacter* sp., *Brachybacterium* sp., *Brevibacterium avium*, *Cornibacterium ammoniagenes*, *Clostridium lituseburense*, *Lactobacillus avarius*, *Jeotgaloccus pinnipedialis*, *Paraliobacillus ryukyuensis*, *Virgibacillus carmonensis* and few other Bacillus species that are responsible for the production of fertilizer from poultry litter. Wet and dry poultry litter samples exhibited the presence of bacterial strains such as *Aerococcus viridans*, *Atopostipes suicloacalis*, *Bacillus hackensackii*, *Brevibacterium avium*, *Corynebacterium ammoniagenes*, *Facklamia sourekii*, *Jeotgalicoccus* sp., *Salinicoccus halodurans*, *Staphylococcus arlettae*, *Staphylococcus cohnii* and *Virgibacillus marismortui* in which *Staphylococcus*, *Salinicoccus*, *Virgibacillus*, *Jeotgalicoccus*, *Facklamia*, *Brevibacterium* and *Bacilli* were found to be dominant. In addition to these, fungal species such as *Candida* sp., *Penicillium pimiteouiense*, *Penicillium decumbens*, *Aspergillus sydowii* and *Euroteum amstelodami* were also identified from the above samples [43]. Food borne bacterial pathogens namely *E. coli*, *Salmonella*, *Campylobacter*, *Staphylococcus*, *Clostridium*, *Listeria*, *Actinobacillus* and *Mycobacterium* were also frequently detected in poultry litter [44].

## 5. Soil Amendment

The manure from chicken, pigs and cows are being used not only for agricultural purposes but also as a feedstock for biogas production. Numerous studies have revealed the various ways how poultry manure prove useful. The addition of poultry litter to tall fescue, orchard grass, Bermuda grass has led to increase in dry matter production [45]. Poultry manure has also been used for the restoration of soil polluted by mining and industrial activities. Poultry manure application increases organic matter and soil moisture contents and water-holding capacity, that in turn improve soil conditions by effecting the indigenous microbial activity enhancing maize yields (Table 1) [46].

### 5.1. As Animal Feed

Poultry litter, either standalone or when mixed with feed grains, is a valuable feed for cattle and fish [47]. However, on the other hand, this practice has raised serious concerns on the safety of using animals waste, with respect to pathogen and disease problems. Cu toxicity has been found to be a major concern especially in sheep fed with poultry manure, more so in sheep since they are less tolerant to high dietary levels of Cu than other livestock species [48,49]. Poultry litter has been used in the United States as animal feed for over 40 years. The specifications for use of poultry litter as an animal feed and the variations in some of its characteristics have been reported [46].

### 5.2. As Fuel

Poultry litter is also being used as fuel in cooking and lighting. It can be burnt directly to produce heat. In many countries around the globe, poultry waste is converted into biogas, whose main constituent is methane, 60%, carbon dioxide 38%, water vapor, hydrogen and hydrogen sulfide. Biogas can be used for the internal combustion engine of farm tractors to generate electricity. Poultry litter combustion has also received major attention in order to produce heat and electricity at large centralized facilities [50]. In the pursuit for generating electric power from renewable ‘green’ sources, a number of cities in the States have turned to thermal conversion of biomass. For example, Minnesota produces over two million tons of turkey and broiler waste—the fuel for ‘poultry power.’ Now some Minnesota turkey farmers are working with a British company (Fibrowatt, Langhorne, PA, USA) that built a manure-powered power plant in central Minnesota (http://fibrowattusa.com). The plant burns nearly half a million tons of poultry litter every year, generating 50 MW of electricity that will be sufficient to supply 40,000 households. The major constraint with the use of poultry litter is the high moisture content and the odor it produces during storage and composting.

## 6. Composting Process of Poultry Manure

Organic waste composting is a microbially mediated biooxidative process that yields a stabilized final product without pathogens, phytotoxic compounds and plant seeds that affect plant growth and productivity through mineralization and partial humification of the organic matter [10]. During the initial phase of the composting process, the simple organic carbon compounds are easily mineralized and metabolized by diverse groups of microorganisms such as bacteria, fungi and micro arthropods to produce CO_2_, NH_3_, H_2_O and organic acids. Once the mesophilic microorganisms encounter organic material, they self-assemble into self-insulating masses, accelerating their growth and metabolism resulting in a rapid increase of temperature that limits mesophilic bacterial growth and stimulates the thermophilic bacterial growth [51]. This extensive microbial metabolic process generates enormous heat energy that raises the temperature of the pile and consequently reduces the volume of the poultry litter wastes [41,52]. Thermophilic composting conditions have resulted in the complete annihilation of lethal gram- negative enteric and pathogenic poultry viruses [53].

## 7. Factors Affecting Composting Process

Successful composting totally relies on microbial decomposition process which in turn depends on various factors such as moisture content, pH, particle size of the poultry litter, compost duration, free air space at which the optimal microbial metabolism is being performed and moreover to the core carbon:nitrogen (C:N) ratio of the poultry litter [54]. It has been reported that the best compost was achieved only at optimum levels of the following factors i.e., carbon:nitrogen (C:N) ratio (between 15 and 25), moisture content (between 40 and 60%), pH (between 5 and 12) and greater than 30% free air space at which the optimal microbial metabolism is being performed [55,56]. Even though the pH and temperature are crucial factors to consider in the composting process, their control had not been usually applied in practical composting operations in full-scale composting systems [57,58,59]. However, the nutrient factor—the C:N ratio—had often been studied as the main factor that controls the poultry composting process. Although, the composting process is carried out under a broad range of initial C/N ratios, (11 to 105) which generally depend on the starter materials [60,61]. Extremely low and high C:N ratios of the compost material yielded low-quality compost with inferior qualities [16,62]. Different co-composting materials were also used to enhance the C:N ratio as well as to reduce the N loss. When the poultry litter was co-composted with biochar, the N losses were reduced up to 25% by lowering the ammonia concentration up to 64% [63]. The co-addition of poultry litter with olive mill waste and mineral rich wastewater as a co-composting material yielded a compost with superior qualities i.e., free of phytotoxicity and no negative impacts on soils [64]. Poultry litter co-composting with various substrates such as wood shaving, excess feed and feathers resulted in compost containing stable organic matter [16]. In addition to that, when pineapple leaves and chicken slurry were used as the initial co-composting material with poultry litter, the compost resulted in an odorless final product with low heavy metals content and substantial amounts of nutrients [65]. Recently, coir pith waste and sugarcane tops are being used as efficient co-composting material of poultry litter [66,67]. Thus, the effect of most of the crucial factors that contribute towards the poultry litter composting processes have been evaluated. However, the role of antibiotics in the composting process of poultry litter which is yet another crucial factor remains overlooked and sidelined. This factor is able to directly affect the microbial community that initiates the primary decomposition process and microbial succession during the composting process.

## 8. Antibiotics Used in Poultry

Antibiotics (as therapeutic agents and as growth-promoting antibiotics (GPAs)) are used in the poultry industry for disease prevention and growth promotion. Their use has been tremendously increased from the 1950s in order to enhance growth, feed efficiency and reduce mortality in broiler production [68,69,70,71]. Owing to frequent controversies regarding the impact of antibiotics used in livestock affecting human health, various public organizations such as The World Health Organization, the American Medical Association and the American Public Health Association have urged a ban on growth-promoting antibiotics (GPAs). They argue that their use leads to increased antibiotic-resistant infections in humans [72,73]. However, the regular inclusion of antibiotics as feed additives to promote growth in the diets of commercial poultry breed still continues as a standard practice in developing and underdeveloped countries. Van Boeckel et al. [74] estimated that the global average annual consumption of antimicrobials per kilogram of poultry animals produced during 2010 was 148 mg kg^−1^. This was found to be much higher than the consumption in cattle, and the study further predicted that the global antimicrobial consumption in the livestock industry will alarmingly increase to 67%, from 63,151 ± 1560 tons to 105,596 ± 3605 tons by 2030.

A vast diversity of antibiotics have been administered in the poultry industry as feed additives as antibiotic growth promoters (AGPs) and also to treat the birds against pathogenic diseases. Table 2 summarizes the various classes of antibiotics such as ionophores, glycopeptide antibiotics, orthosomycins, diterpene antibiotics, tetracyclines, aminoglycosides, cyclic peptides, lincosamides, polymyxins, fluoroquinolone, sulfonamides, macrolides, streptogramins, quinolones, diaminopyrimidines and β-Lactams that were and are being used for poultry including, chickens, turkeys, ducks, ostriches, pigeons, pheasants and quails.

Ionophores are ion-carrier molecules that facilitate ion transport across lipid membrane due to their lipid-soluble nature and reversible ion binding ability. The ionophores used in the poultry industry as feed additives are predominantly obtained from *Streptomyces* and other fungal species as their fermentation products [122].They are used extensively as anticoccidials and they act against the protozoan parasite *Eimeria* species and Gram-positive bacteria in broiler chicken, turkeys, pheasants and quails. They exhibit antimicrobial activity by binding to the cell wall (ergosterol) and facilitate extensive leakage of cellular K^+^ ions which results in acidification and death of the poultry pathogens. In the poultry industry ionophore antibiotics such as bambermycin/flavomycin, maduramicin, narasin, monensin, lasalocid, salinomycin, semduramicin and nystatin were used as feed additives [75,76,77,78].

Glycopeptide antibiotics fall under the category of antibiotics that have glycosylated cyclic or polycyclic nonribosomal peptides, and interfere with the cell wall and protein synthesis of enterococcal pathogens. However, their application was extremely limited for the fear of creating methicillin-resistant strains of *Staphylococcus aureus* (MRSA) in poultry chicken and their presumable infectivity in humans. Therefore, in most of the developed countries the usage of glycopeptides antibiotics had been banned [85]. The FDA Center for Veterinary Medicine (FDA-CVM) also issued an order following the European ban on vancomycin in 1997 prohibiting the extra-label use of all glycopeptides in food animals [123]. However, the usage of such antibiotics is yet to be strictly monitored in under-developed countries.

Avilamycin—a drug from the orthosomycin family (due to its orthoester links with carbohydrates moieties)—is also used as a growth promoter in broiler chickens and turkeys [124]. The drug effectively controls the Gram-positive bacterial community structure in poultry birds through the inhibition of ribosomal function and subsequent protein synthesis in bacteria [116,125]. A diterpene antibiotic namely tiamulin hydrogen fumarate, a semi synthetic derivative of pleuromutilin was found to be effective against different *Mycoplasma*- and *Brachyspira*-related diseases in poultry chickens and turkeys through inhibiting protein synthesis [93,126].

Tetracyclines, are yet another well-known group of antibiotics used to control various groups of disease causing microorganisms in poultries such as gram positive and gram negative bacteria; *Mycobacterium, Mycoplasma, Nocardia, Streptomyces* and *Ureaplasma*. The tetracycline derivatives like chlortetracycline, doxycycline, oxytetracycline, tetracycline are used to control pathogens and thereby aid in improving the feed efficiencies and promote growth in chickens, turkeys, pigeons, ostriches, ducks. They also act through the inhibition of protein synthesis [94,95,96,100]. For treatment of diseases caused by some mycoplasma and Gram-positive anaerobes in poultry chickens, antibiotics from the lincosamides group namely lincomycin, clindamycin and pirlimycin. that interfere with protein synthesis are also used [3,100,110]. Respiratory diseases and gastrointestinal infections caused by *Pseudomonas aeruginosa* in poultry chickens, turkeys and ducks have often been treated with fluoroquinolone group of antibiotics i.e., ciprofloxacin, ofloxacin. Trimethoprim Enrofloxacin, marbofloxacin and difloxacin that block DNA replication [104,105,106]. Macrolides such as azithromycin, clarithromycin, clindamycin, *erythromycin*, roxithromycin, spiramycin, *tylosin* are prevalently administered with feed of layer and broiler chickens against beta-hemolytic *Streptococci*, *Pneumococci*, *Staphylococci* and *Enterococci*, *Mycoplasma*, *Mycobacteria*, some *Rickettsia* and *Chlamydia* infections and their mode of action was also through the inhibition of protein synthesis [110,111,112,113]. Antibiotics belonging to the streptogramins group and quinolones namely virginiamycin and nosiheptide, specifically act against Gram-positive bacterial infections by blocking protein synthesis of those pathogens in chickens and turkeys [86,114,115,116]. Diaminopyrimidines such as Trimethoprim, aditoprim, baquiloprim, ormetoprim often act as bactericidal agents in the presence of sulfonamides against Gram-positive and many Gram-negative bacteria through the inhibition of nucleic acid synthesis [117]. The traditional antibiotic group called β-Lactams was observed to be used in poultries. Various derivatives of penicillins such as amoxicillin, ampicillin, benzylpenicillin, cloxacillin, dicloxacillin, flucloxacillin, methicillin, mezlocillin, nafcillin, oxacillin, piperacillin, phenoxymethylpenicillin are predominantly used for controlling infections such as Necrotic enteritis, ulcerative enteritis and intestinal spirochetosis in poultry caused by *Enterococcus faecalis.* The antibiotics inhibit cell wall synthesis [118,119,120,121]. Aminoglycosides antibiotics such as gentamicin, tobramycin, amikacin, streptomycin, kanamycin, neomycin, spectinomycin, have also been used invariably in broiler chickens, ducks and turkeys. These drugs exhibit great control over pathogenic Gram-positive and negative bacteria through inhibiting bacterial protein synthesis [97,98,99]. In addition to that, cyclic peptide antibiotics, bacitracin and its derivative zinc bacitracin, which inhibit the bacteria through blocking protein synthesis have been used to control both Gram-positive and negative bacteria that causes diseases in layer chickens, turkeys, ducks and pigeons [3]. polymyxins such as polymyxin B, colistin (polymyxin E) have also been prevalently used to control disease caused by most Gram-negative bacteria in chickens [102,103,127].

Thus, the commercial poultry farming is adequately supplied with umpteen numbers of antibiotics in large quantities and therefore it is believed and established that a large portion of the unmetabolized antibiotics could possibly be excreted out and stay longer in poultry litter which would not only act against the poultry associated microflora but also work against common beneficial microflora that involve in the composting process. The concentration of antibiotics administered, and the residual antibiotics excreted in poultry litter have been extensively reviewed. It is a well-known fact that almost all the antibiotics used in poultry industry are minimally metabolized and excreted via urine and feces of birds, for instance tyrosine used in poultry chicken, is excreted primarily in urine to a larger extent and the remaining through feces [128,129]. Some aspects have been addressed in a review by Goetting et al. 2011 [130] on the pharmacokinetics of drugs used for layering chicken. Most of the antibiotics used in poultry are concentrated in poultry litter unmetabolized to a major extent, another review on global antibiotic poultry antibiotic usage and their in-tissue assimilation by Van Boeckel et al. [74] is also informative. The data of antibiotic residues of poultry chicken in various nations was reviewed by Mauz et al. 2018 [131]. These existing reviews report the concentrations and antibiotic residues, in chicken meat and not in chicken litter. All these are restricted to residues in the meat. There is no information or detailed study on the concentration of the antibiotics in the poultry litter and more so almost no information on the residual antibiotic concentration in the poultry compost (the pretreated poultry litter used for agricultural purposes). The subsequent residual antibiotic concentrations released to the soil when the compost has been used in the soil and thereafter the residual concentrations carried over to the plants/ agricultural crops have not been studied. There are many gray areas in this area. This review identifies this lacuna and emphasizes that this is the need of the hour. An enlightenment in this area could bring about an awareness on the environmental and toxicological impact of this flow.

## 9. Future Prospects

After contamination of niches with antibiotics, these have become one of the vital ecological factors in any of the ecosystems that influence the evolution of the community structure [132]. Subsequently, the community structural changes in turn affect the ecosystem functions such as biomass production and nutrient transformation [133,134]. Therefore, there is no doubt that the poultry litter, before composting, contains a large quantity of a wide variety of antibiotics and its entry into the food chain at various levels needs to be closely monitored. Furthermore, it has been reported that the potential antibiotic effect that influence the community structure stays for a prolonged period (e.g., sulfadiazine up to 175 days) [135]. Although many research works concerning the poultry compost preparation have taken various factors such as C/N ratio, moisture content etc., into consideration during the optimization of the composting process, the role of antibiotics, in the poultry composting process has not been studied so far. Composting involves the involvement of the activity of the inherent microbes in the litter, so antibiotics in the poultry litter affecting the microbial diversity of the poultry litter will crucially affect the successful composting process. Therefore, studying the fate of antibiotics in the poultry litter is a crucial prerequisite since it invariably affects human beings either directly through consumable poultry based products or indirectly through inhibiting the agriculturally beneficial microbial population in the poultry waste. Furthermore, the antibiotic load in poultry litter would certainly affect the biooxidative process and thus compost maturity would be largely delayed, which is yet another main factor that needs to be considered. All these gray areas specified, and issues raised, are yet to be addressed. This review hopes that a sensitization and awareness triggering research in these associated areas will ignite.

**Figure 1:**
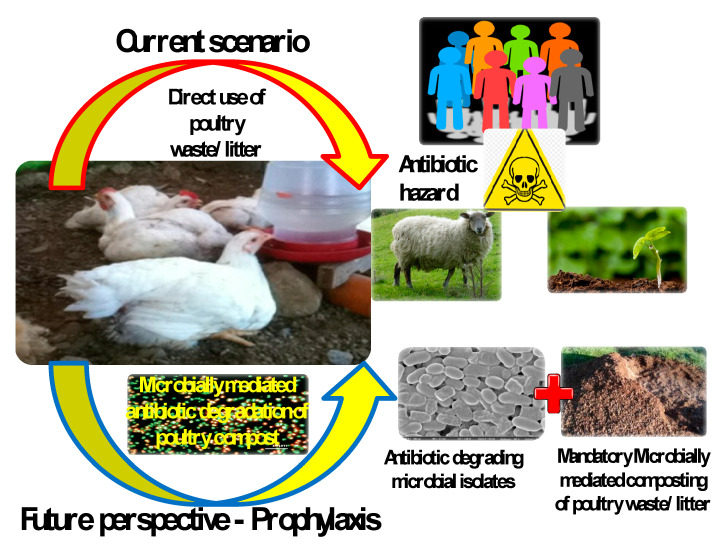
Schematic emphasizing the futuristic environmentally friendly and promising microbially accelerated poultry waste/litter composting.

The other prophylaxis is through the deliberate addition of microbial isolates that hold a reputation as antibiotic degraders and hydrolytic enzyme producers, to the poultry litter at the very initial stage of composting for accelerating the composting process. This would ideally degrade the antibiotics in the poultry litter and create a congenial environment for the composting process to take its course. Figure 1 sums up the current and the future prospects of poultry litter management. This review emphasizes on the introduction of antibiotic degraders as a mandatory prerequisite for accelerated microbially mediated composting of poultry litter, for effective composting and for environmentally friendly antibiotic free accelerated production of poultry compost for agricultural use.

## 10. Conclusions

This review has questioned the fate of residual antibiotic in poultry waste that has the potential to negatively impact microbial communities that are involved in microbial mediated poultry litter composting processes. The incorporation of antibiotic degrading environmental isolates in poultry litter at the initial stage of composting in order to accelerate the process is addressed in this review as a future perspective. This review aims at creating an awareness and sensitization in this direction.

## Figures and Tables

**Table 1:** Characteristics of processed poultry waste-based animal feeds (FDA, 2009).

Category	Description	Moisture Content (%)	Crude Protein%	Crude Fiber%	Ash%	Feathers%
Dried poultry waste	Feces	>15	<18	>15	>30	>1
Dried poultry waste	Void of part or all of crude protein and non-protein nitrogen, feces	>15	<11	>15	>30	>1
Dried poultry litter	Feces and floor litter	>15	<18	>25	>20	>4

**Table 2:** Antibiotics used in poultry industry for the treatment of microbial diseases and also as feed additives as antibiotic growth promoters (AGPs).

Antibiotics Used in Poultry	Activity Against Microbial Pathogens	Mode of Action	Used for Poultry Species	Reference
**Ionophores**				
flavomycin/bambermycin/flavophospholipol, moenomycin	Gram-positive bacteria	Inhibition of cell wall synthesis	broiler chicken and turkeys	[75,76]
maduramicin, narasin and monensin	protozoan parasite (*Eimeria* sp.)	Cell wall disruption	broilers	[77,78]
lasalocid	protozoan parasite (*Eimeria* sp.)	Cell wall disruption	broilers and replacement pullets, turkeys, pheasants and quails.	[79]
salinomycin	protozoan parasite (*Eimeria* sp.)	Cell wall disruption	chicken and turkey	[80]
semduramicin	protozoan parasite (*Eimeria* sp.)	Cell wall disruption	chicken and turkey	[81]
nystatin	*Candida albicans*	Cell wall disruption	chicken and turkey	[82]
**Glycopeptide antibiotic**				
avoparcin	Gram-positive bacteria	Inhibition of cell wall synthesis	Broiler chicken and turkeys	[78,83,84]
vancomycin, teicoplanin, avoparcin, ardacin	Gram-positive bacteria and *Actinomycetes* such as *Streptomyces orientalis* (vancomycin), *Nocardia actinoides* (Actinoidin)	Inhibition of cell wall synthesis	Broiler chickens and Turkey	[85,86,87]
**Orthosomycin**				
avilamycin	Gram-positive bacteria	Inhibition of protein synthesis	Broiler chicken and turkeys	[88,89,90,91,92]
**Diterpene antibiotics**				
tiamulin hydrogen	*Mycoplasma* and *Brachyspira*	inhibition protein synthesis	Broilers chickens	[93]
**Tetracyclines**				
chlortetracycline, doxycycline, oxytetracycline, tetracycline	Gram-positive and Gram-negative bacteria, *Mycobacterium, Mycoplasma, Nocardia, Streptomyces* and *Ureaplasma*	inhibition protein synthesis	Turkeys, Pigeons, Ostriches, ducks	[94,95,96]
**Aminoglycosides**				
gentamicin, tobramycin, amikacin, streptomycin, kanamycin, neomycin, spectinomycin	Gram-positive and negative bacteria	Tetracyclines	Broiler chickens, Ducks and Turkeys	[97,98,99]
**Cyclic peptide**				
bacitracin/zinc bacitracin	Gram-positive and Gram-negative Bacteria	Inhibition of Cell wall and protein synthesis	Layers, Turkeys, Ducks, pigeons	[3]
**Lincosamides**				
Lincomycin, Clindamycin and Pirlimycin	*Mycoplasma* and Gram-positive anaerobes	Inhibition of protein synthesis	Chickens	[3,100,101]
**Polymixins**				
polymixin B, colistin (polymyxin E)	Gram-negative bacteria	Inhibition of cell membrane function	Chickens	[102,103]
**Fluoroquinolones**				
ciprofloxacin, ofloxacin. trimethoprim enrofloxacin marbofloxacin and difloxacin	Gram-negative bacteria	Inhibition of DNA replication	Chickens, Turkey and Ducks	[104,105,106]
sulfonamides				
sulfadimidine, sulfamethoxazole.	Gram-positive and Gram-negative bacteria and Protozoa	Inhibition DNA replication	Broiler and Layer Chickens and Turkeys	[107,108,109]
**Macrolides**				
azithromycin, clarithromycin, clindamycin, erythromycin, roxithromycin, spiramycin, tylosin, vancomycin	Gram-positive bacteria, *Mycoplasma*, *Mycobacteria*, some *Rickettsia* and *Chlamydia*	Inhibition of protein synthesis	Layer and broilers Chickens	[110,111,112,113]
**Streptogramins**				
virginiamycin	Gram-positive bacteria	Inhibition of protein synthesis.	Chickens and Turkeys	[86,114,115,116]
nosiheptide		Inhibit Protein synthesis	Broiler chickens	[86,114,115,116]
**Quinolones**				
quinoxalines	Gram-positive Organisms	Inhibition of DNA replication	Chickens and Turkey	[116]
Diaminopyrimidines				
Trimethoprim, aditoprim, baquiloprim, ormetoprim	Gram-positive and many Gram-negative bacteria	Inhibition of DNA replication	Chickens	[117]
**β-Lactams**				
Penicillins: amoxicillin, ampicillin, benzylpenicillin, cloxacillin, dicloxacillin, flucloxacillin, methicillin, mezlocillin, nafcillin, oxacillin, piperacillin, phenoxymethylpenicillin	Gram-positive and many Gram-negative bacteria	Inhibition of cell wall synthesis	Fattening Turkeys Broiler Chickens	[118,119,120,121]
